# Longitudinal Trend in Hospital-Wide Syphilis Testing and Clinical Characteristics of Syphilis Requiring Treatment

**DOI:** 10.3390/pathogens14090892

**Published:** 2025-09-05

**Authors:** Yukiko Takemori-Sakai, Shiori Kitaya, Shigeki Nakaguchi, Tomoko Takayama, Kenichi Takemoto, Hiroyasu Oe, Shigeki Sato, Mika Mori, Hajime Kanamori

**Affiliations:** 1 Division of Clinical Laboratory Medicine, Kanazawa University Hospital, Kanazawa 920-8641, Ishikawa, Japan; shigeki-knz@staff.kanazawa-u.ac.jp (S.N.); takemotoknz@gmail.com (K.T.); h.oe@staff.kanazawa-u.ac.jp (H.O.); s.sato@staff.kanazawa-u.ac.jp (S.S.); morimika@staff.kanazawa-u.ac.jp (M.M.);; 2 Division of Infection Control, Kanazawa University Hospital, Kanazawa 920-8641, Ishikawa, Japan; 3 Division of Medical Oncology, Kanazawa University Hospital, Kanazawa 920-8641, Ishikawa, Japan; 4 Department of Cardiovascular and Internal Medicine, Kanazawa University Hospital, Kanazawa 920-8641, Ishikawa, Japan

**Keywords:** syphilis, *Treponema pallidum* subspecies *pallidum*, rapid plasma reagin, ocular syphilis, uveitis

## Abstract

Globally, syphilis cases are rising, and varied symptoms hinder diagnosis, highlighting the role of serological testing. Comprehensive institutional analysis is needed, as many reports are limited. We retrospectively reviewed 23 persons with syphilis treated at Kanazawa University Hospital (January 2007–December 2023). Of 9145 individuals tested for treponemal (chemiluminescent enzyme immunoassay) and nontreponemal (rapid plasma reagin, RPR) antibodies, diagnoses were based on clinical assessment and serology. Data on history, stage, lesions, and treatment were collected. From 2007 to 2023, antibody testing increased, but treated cases stayed stable (0–4/year). Males comprised 60.9% and females 39.1%. In the treated 23 patients, ophthalmology (30.4%) and dermatology (17.4%) accounted for nearly half of test requests, though cases arose across specialties. Early-stage and ocular syphilis each occurred in 34.8%. Uveitis was the most common lesion (26.1%). Treatment included amoxicillin (69.6%) or penicillin (21.7%). At diagnosis, 78.3% were dual-positive and 21.7% treponemal-only positive. Nine patients (64.3%) had a ≥4-fold RPR titer decline (median 143.5 days); no RPR increases suggested treatment failure or reinfection. This study found stable treated numbers, with ocular syphilis and uveitis frequent. In specialized institutions, clinicians should consider syphilis in persons with varied symptoms across departments, ensuring comprehensive testing and appropriate follow-up.

## 1. Introduction

Syphilis is a sexually transmitted infection (STI) caused by *Treponema pallidum* subsp. *pallidum*. The World Health Organization reported that the estimated number of new syphilis cases increased from 7.1 million in 2020 to 8.0 million in 2022 [1]. In Japan, cases have steadily risen since approximately 2011, decreased between 2019 and 2020 during the coronavirus disease 2019 (COVID-19) pandemic, and increased again since 2021. In 2022, 13,258 new syphilis cases were reported, the highest since national surveillance began in 1999 under the Act on the Prevention of Infectious Diseases and Medical Care for Patients with Infectious Diseases (Infectious Diseases Control Law) [2]. The pandemic-related decrease likely reflected reduced transmission opportunities from behavioral restrictions, as well as underdiagnosis and undertesting due to laboratory closures and fewer clinic visits. However, these factors contributed to delayed diagnosis and treatment, facilitating further spread [3,4,5,6]. Syphilis is typically diagnosed when patients present with lesions such as ulcers, hard chancres, or rashes, though rare manifestations, such as gastric ulcers, flat elevated gastric lesions, or palate perforation, can also occur [7,8]. In addition, nonspecific findings such as trunk rash can be misdiagnosed as lichen planus or viral exanthem if syphilis is not considered [9,10]. Thus, diagnosis remains challenging, even for experienced clinicians, due to its diverse clinical manifestations. Uveitis is also a nonspecific finding associated with syphilis. Although syphilitic uveitis is generally considered rare, its incidence has been reported to increase in recent years in parallel with rising syphilis cases [11]. Because syphilitic uveitis can cause severe vision loss, its impact on patients’ lives is substantial [12]. To ensure a favorable visual prognosis, it is essential to minimize the time from the onset of ocular symptoms to the initiation of treatment [13,14].

Most syphilis cases are diagnosed using serological tests. Rapid plasma reagin (RPR) detects cardiolipin antibody (nontreponemal antibody), and *T. pallidum* particle-agglutination test, enzyme immunoassay (EIA), chemiluminescence immunoassay (CIA), and chemiluminescent enzyme immunoassay (CLEIA) detect treponema-specific antibodies. Understanding the clinical and laboratory characteristics of syphilis is essential for accurate diagnosis and effective treatment. Although several studies on syphilis are case reports or describe individual manifestations, most are limited to specific disease stages. Some reports from university hospitals suggest that late latent syphilis is the most common stage and that oropharyngeal symptoms are relatively frequent; however, studies encompassing all patients with syphilis at a single medical facility are scarce [15,16]. To address this gap, we conducted a comprehensive analysis of the clinical and laboratory characteristics of patients with syphilis diagnosed and treated at a single university hospital. This study provides a hospital-wide, longitudinal analysis of syphilis cases across multiple departments, including rare manifestations such as ocular and neurosyphilis, and examines real-world diagnostic and follow-up challenges. Furthermore, it offers insights that may inform future improvements in clinical decision-making.

## 2. Materials and Methods

### 2.1. Study Population and Data Collection

In total, 9145 patients underwent treponemal and nontreponemal antibody testing simultaneously at Kanazawa University Hospital from 1 January 2007, to 31 December 2023. Of these, patients diagnosed with syphilis requiring treatment were included in this study after excluding those whose treponemal antibody and RPR were negative, whose antibody test was false positive, with past infection, maternal antibody transfer, or refusal of treatment. Age, sex, ordering department, treponemal antibody test results, and qualitative and quantitative nontreponemal antibody test results were extracted using CLINILAN (A&T Corporation, Kanagawa, Japan), a clinical laboratory information system enabling real-time monitoring of laboratory test status and data extraction. Medical records (MegaOakHR, NEC Corporation, Tokyo, Japan) of treponemal or nontreponemal-positive patients were reviewed to identify those diagnosed by physicians as requiring antimicrobial therapy or other treatments based on physical examination, patient history, and serological results. For these patients, comorbidities, history and complications of other STIs, syphilis stage, lesion type, and treatment were also obtained from medical records. This group included three patients (13.0%) diagnosed at our hospital but treated elsewhere.

### 2.2. Classification of Syphilis

We classified syphilis into ocular syphilis, neurosyphilis, early syphilis (primary, secondary, and early latent), and late syphilis (tertiary and syphilis of unknown duration) [17,18,19]. Ocular syphilis was diagnosed in patients with positive treponemal antibodies and ocular lesions unexplained by another etiology [18,20]. Neurosyphilis was diagnosed comprehensively based on cerebrospinal fluid examination (e.g., cell count), syphilis antibody tests, and neurologic symptoms [19]. Early latent syphilis was defined as an asymptomatic case in which the opportunity for infection was identified within a year of examination. Syphilis of unknown duration was defined as an asymptomatic case in which the time of infection could not be determined [17,19].

### 2.3. Treponemal and Nontreponemal Antibody Tests

CLEIA (FUJIREBIO Inc., Tokyo, Japan) was used for the treponemal antibody test, and RPR (SEKISUI MEDICAL Co., Ltd., Tokyo, Japan) was used for the nontreponemal antibody test. Both tests were performed according to the procedures described in each manufacturer’s instructions.

### 2.4. Change in the Rapid Plasma Reagin Titer After Treatment

We examined changes in the RPR titer after treatment in 14 patients for whom pretreatment RPR titers were available and for whom titers were measured at least twice. The most recent RPR titer on 31 December 2023, was compared with the pretreatment titer. For nine patients whose titers decreased two-fold or more, the number of days to achieve this reduction was recorded. One patient with an eight-fold titer decrease was excluded because the patient was transferred to another hospital, resulting in a gap in RPR monitoring.

### 2.5. Symptom and Lesion After Treatment

Recovery was defined as improvement of both symptoms and lesions. None was defined as latent syphilis that was asymptomatic before treatment and remained asymptomatic after treatment. Consistency was defined as no improvement of symptoms and/or lesions after treatment.

### 2.6. Follow-Up

Appropriate follow-up was defined as periodic RPR titer measurement for 1 year in RPR-positive cases and periodic treponemal antibody measurement for 1 year in RPR-negative cases [18].

## 3. Results

### 3.1. Status of Antibody Tests and Diagnosis of Syphilis

The treponemal antibody test and RPR were simultaneously performed in 9145 patients at Kanazawa University Hospital from 2007 to 2023. In 9145 patients, neurology (*n* = 2718, 29.7%) and ophthalmology (*n* = 2031, 22.2%) accounted for approximately half of all test requests, followed by obstetrics and gynecology (*n* = 1425, 15.6%), rheumatology (*n* = 634, 6.9%), dermatology (*n* = 494, 5.4%), and others (*n* = 1843, 20.2%). Treponemal antibody positivity rates in neurology, ophthalmology, obstetrics and gynecology, rheumatology, and dermatology were 1.4% (*n* = 38), 1.2% (*n* = 24), 0.6% (*n* = 9), 1.1% (*n* = 7), and 2.2% (*n* = 11), respectively. The corresponding RPR positivity rates were 0.8% (*n* = 23), 1.2% (*n* = 25), 0.7% (*n* = 10), 1.3% (*n* = 8), and 2.2% (*n* = 11). Rates of patients actually treated were 0.1% (*n* = 2), 0.3% (*n* = 7), 0.1% (*n* = 2), 0.2% (*n* = 1), and 0.8% (*n* = 4), respectively. Overall, the sensitivity, specificity for detecting treated patients, and positivity rate by treponemal antibody testing were 100% (*n* = 23/23), 98.9% (*n* = 9022/9122), and 1.3% (*n* = 123/9145), respectively, and for RPR were 78.3% (*n* = 18/23), 98.9% (*n* = 9024/9122), and 1.3% (*n* = 116/9145) (Table 1).

**Figure 1:**
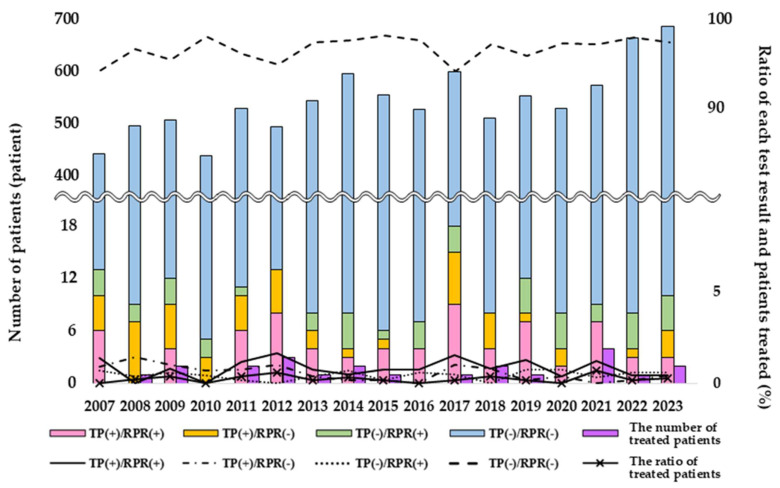
Changes in the number and ratio of antibody tests, test results, and patients treated. The bar graph depicts the number of patients who underwent both the treponemal antibody test and RPR or received treatment. The line graph illustrates the ratio of each test result and of patients treated. The wavy line indicates the omission of numerical values. The number of antibody tests for syphilis increased; however, the number of treated patients remained unchanged (0–4 patients/year). TP, treponemal antibody test; RPR, rapid plasma reagin; +, positive; -, negative.

From 2007 to 2023, the number of antibody tests increased overall, with a temporary decline in 2020 followed by a rise from 2021 (Figure 1).

**Figure 2:**
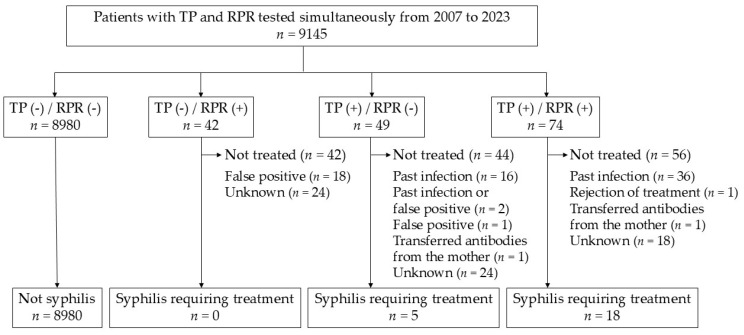
Patient selection criteria used in this study. We included 23 patients with syphilis diagnosed by physicians as requiring treatment from among 9145 patients who simultaneously underwent the treponemal antibody test and RPR. TP, treponemal antibody test; RPR, rapid plasma reagin; +, positive; -, negative.

Although the number of antibody tests increased, the number of cases negative for both treponemal antibody and RPR also rose, and the number of treated patients (0–4 patients/year) remained unchanged. This study included 23 patients diagnosed with syphilis who required treatment (Figure 2).

### 3.2. Characteristics of the Study Population

The clinical characteristics of the treated patients are presented in Table 2.

The treated patients included 14 males (60.9%) and nine females (39.1%). The median (interquartile range [IQR]) ages were 51.5 years (46.8–57.0) for males and 44.0 years (35.0–73.0) for females. Many males were in their 50s, whereas females were treated across a wider age range. For early syphilis, the median (IQR) ages were 58.0 years (46.0–67.0) for males, while females were younger at 17.0 years (17.0–30.5). In the treated 23 patients, ophthalmology (*n* = 7, 30.4%) and dermatology (*n* = 4, 17.4%) accounted for approximately half of antibody test requests, but syphilis cases were observed across various departments, particularly for late syphilis. Early and ocular syphilis were the most common stages, each observed in eight (34.8%) patients, followed by late syphilis in five (21.7%) and neurosyphilis in four (17.4%). Three (13.0%) patients had both ocular and neurosyphilis; three (37.5%) patients with ocular syphilis had neurosyphilis, and three (75.0%) patients with neurosyphilis had ocular syphilis. The most common lesion was uveitis (*n* = 6, 26.1%), followed by optic nerve lesions (*n* = 5, 21.7%). Sixteen (69.6%) patients were treated with oral amoxicillin, four (17.4%) received 1,500 mg/day for 4 weeks, and five (21.7%) for at least 5 weeks. Five (21.7%) patients received intravenous penicillin, three (13.0%) at 24 million units/day for 2 weeks, and one (4.3%) at 18 million units/day for 2 weeks. Half of the patients with ocular syphilis (*n* = 4; 3 of whom also had neurosyphilis) and all patients with neurosyphilis (*n* = 4) received intravenous penicillin at 24 million units/day or 18 million units/day for 2 weeks. In one of these patients, penicillin administration was discontinued due to phlebitis. The remaining patients with syphilis were treated with oral amoxicillin. After treatment, 10 (43.5%) patients improved in symptoms and lesions, whereas three (13.0%) had no improvement in symptoms or/and lesions. Three patients with concomitant ocular syphilis and neurosyphilis, neurosyphilis, and tertiary late syphilis had residual low vision from optic atrophy, cognitive decline from general paralysis, and lightning pain from tabes dorsalis. Of 15 patients followed at our hospital after treatment, eight (53.3%) were followed appropriately. Notably, many early syphilis cases were not followed appropriately, occurring in four (66.7%) patients. A list of the treated 23 patients is presented in Table S1.

### 3.3. Results of Treponemal Antibody Test and RPR and Therapeutic Effectiveness

Table 3 presents the results of the treponemal antibody test and RPR. Eighteen (78.3%) cases were RPR-positive when the treponemal antibody test was performed, and five (21.7%) were negative. These five cases included early syphilis (*n* = 2), late syphilis (*n* = 2), and ocular syphilis (*n* = 1). One early syphilis case (no. 5) converted from RPR-negative to positive when retested 9 days later. After treatment, the RPR titer decreased in 11 (78.6%) patients, remained unchanged in two (14.3%), and increased two-fold in one (7.1%). Nine (64.3%) cases showed a four-fold or greater decrease in RPR titer: early syphilis (*n* = 3), ocular syphilis (*n* = 5), and neurosyphilis (*n* = 3), with two involving ocular–neurosyphilis coinfections. One patient (no. 5) became negative from a 1:1 RPR titer after treatment and was considered successfully treated based on a concurrent decrease in treponemal antibodies. The proportions of successful antimicrobial therapy (four-fold or greater RPR decrease and 1:1 to negative) were 80.0% (*n* = 4) for early syphilis, 83.3% (*n* = 5) for ocular syphilis, and 75.0% (*n* = 3) for neurosyphilis, but 0% for late syphilis. The median time to achieve a four-fold or greater RPR decrease was 143.5 days (IQR, 98.5–169.5; range, 49.0–186.0).

## 4. Discussion

### 4.1. Comparison with Japanese Syphilis Trends

In Japan, the number of syphilis cases has continued to increase since approximately 2011, decreased between 2019 and 2020 during the COVID-19 pandemic, and increased again since 2021 [2]. In our study, the number of antibody tests increased between 2007 and 2023, declined in 2020, and rose again from 2021. We consider the 2020 decrease in antibody tests to be the result of the COVID-19 pandemic that began spreading in Japan that year [21]. Although antibody testing at our hospital increased, the number of syphilis diagnoses requiring treatment remained unchanged, differing from the national trend. We believe this is because our highly specialized university hospital receives referrals for cases difficult to diagnose or treat elsewhere. Therefore, unlike general hospitals, there was a selection bias in the syphilis cases we observed. However, the median (IQR) age for early syphilis was 58.0 years (46.0–67.0) for males and 17.0 years (17.0–30.5) for females, indicating a predominance of younger females. This reflects the national trend in Japan, where syphilis occurs across men of all ages, particularly those in their 20s–40s, while women are predominantly in their 20s [2].

### 4.2. Sensitivity, Specificity, and Positivity Rate in Antibody Testing

The sensitivity and specificity for detecting treated cases were 100% and 98.9% for the treponemal antibody test, and 78.3% and 98.9% for RPR, respectively, indicating the utility of antibody testing for syphilis diagnosis. However, the antibody positivity rate was low at 1.3% for both tests, similar to the previously reported 1.45% [22]. In Japan, routine admission screening for syphilis to prevent bloodborne transmission continues; however, we consider its clinical significance is low because bloodborne syphilis transmission is rare, the testing increases costs, and incorrect interpretation of biological false positives, especially for RPR and treponemal antibodies that often remain positive after appropriate treatment, may lead to unnecessary treatment [22,23]. That said, there have been cases where active syphilis was detected on admission screening, and one case of neurosyphilis readily diagnosed when psychiatric symptoms were present [22,24]. In university hospitals, where many undiagnosed cases present, lowering barriers to testing when lesions do not rule out syphilis remains desirable.

### 4.3. Department of Patients with Syphilis Visit

Syphilis is generally diagnosed in urology, dermatology, and obstetrics and gynecology [25], but in this study, ophthalmology and dermatology accounted for approximately half of the cases, with various other departments represented. Late syphilis was observed across multiple departments. A report from a university hospital indicated that internal medicine and dermatology accounted for more than half of first-visit patients; however, syphilis was also identified in departments such as surgery, infectious diseases, and obstetrics and gynecology [26]. Another report described unexpected syphilis diagnoses in orthopedics, internal medicine, surgery, and otolaryngology through preoperative admission screening [22]. In highly specialized medical institutions, such as university hospitals, the possibility of encountering syphilis across multiple specialties should be considered in daily care. A distinctive feature of this study is the large number of ocular syphilis cases with uveitis in ophthalmology. Ocular syphilis frequently presents as uveitis [19]. Our ophthalmology department actively considers syphilis in patients presenting with uveitis and requests antibody tests in a relatively high number of cases. Test requests from ophthalmology accounted for 22.2% of all cases, higher than dermatology, urology, or obstetrics and gynecology, resulting in more syphilis cases in ophthalmology than in other departments. In contrast, neurology submitted the most test requests (29.7%) but treated only two patients, a low number considering the request volume. Dementia is the most common symptom of neurosyphilis [27], and in our neurology department, antibody tests are often ordered for patients with dementia or cognitive decline. Most cases of these symptoms are commonly associated with Alzheimer’s disease dementia or vascular dementia and are rarely caused by syphilis [28]. Therefore, the neurology department may have had relatively few treated patients compared with the number of test requests. However, as some patients with syphilis develop dementia or cognitive decline, careful evaluation of such presentations should always include consideration of syphilis.

### 4.4. Syphilitic Uveitis

In this study, ocular syphilis was as common as early syphilis, with uveitis being the most frequent lesion. Syphilitic uveitis is generally considered rare but has been reported to increase alongside rising syphilis cases in recent years [11]. It has been described in uveitis cases of unknown cause, in those with symptoms unresponsive to treatment, and who were referred to specialized hospitals [13,29]. In our study, all patients with uveitis were referred from other clinics, supporting these observations. The frequent detection of syphilis in uveitis cases is characteristic of university hospitals and suggests that the possibility of syphilis should be considered in uveitis evaluations at such institutions. Syphilitic uveitis can seriously impact visual outcomes; in one study, 19 (23.5%) of 81 eyes had ≤1.0 (20/200) vision after treatment [12]. Another study showed that initial misdiagnosis, lower baseline visual acuity, and a delay of >12 weeks between diagnosis and treatment were linked to poorer vision after 6 months [13,14]. These findings highlight the importance of minimizing time from ocular symptom onset to treatment initiation. At our hospital, among five cases of syphilitic uveitis with follow-up data, one (no. 16) had decreased vision from cataract and epiretinal membrane, while four achieved final visual acuity > 0 (20/20), indicating appropriate management from consultation to treatment. Syphilitic uveitis is more often associated with posterior uveitis and panuveitis [13,14]. Particularly in unexplained posterior uveitis or panuveitis, it is important to suspect syphilis and actively perform testing at a lower threshold. Rapid, accurate diagnosis and appropriate therapy can markedly improve prognosis.

### 4.5. Cases with Treponemal Antibody Test-Positive and RPR-Negative Results

At diagnosis, five patients were positive for treponemal antibody tests but negative for RPR. This pattern can occur in previously treated syphilis, long-standing untreated syphilis, prozone reaction, and early syphilis [30]. The RPR titer often declines rapidly after treatment, but may also decline more slowly without treatment. Three patients (nos. 3, 9, 12), who were RPR-negative, had already been treated for syphilis before visiting our hospital, likely due to antimicrobial therapy. In one patient (no. 5), the RPR converted from negative to positive after 9 days, suggesting a weakly positive result in early infection; the initial RPR titer was 1:1. A study comparing RPR and treponemal antibody tests (e.g., EIA, CIA) found lower RPR sensitivity, particularly for early syphilis [31,32]. In patient no. 5, the treponemal antibody was presumed positive before RPR due to early infection. A prozone reaction, false-negative RPR from antibody excess, has been reported with titers from 1:8 to 1:512 [33]. In patient no. 5, the 1:1 RPR excluded prozone. Another patient (no. 20) had ocular syphilis, in which human immunodeficiency virus (HIV) was diagnosed at the same time. Although the apparent reason for the RPR-negative result is unclear, a quantitative RPR test was not performed in this patient, and the prozone reaction could not be ruled out. False negatives because of prozone reactions can lead to misdiagnosis, delays in diagnosis, and treatment. A case of congenital syphilis in a 3-month-old infant was misdiagnosed as an upper respiratory tract infection when the infant showed symptoms, because his mother’s prenatal examination showed a false-negative RPR because of a prozone reaction, which delayed the diagnosis and initiation of treatment. In this report, after the infant was diagnosed with congenital syphilis, his mother’s RPR test was performed again with the same blood sample obtained at the time of the prenatal examination, which was initially negative. After requesting that the laboratory dilute the sample, the RPR was positive [34]. Prozone reactions have also been reported in cases of HIV coinfection. In these cases, the physician requested RPR again with higher dilution, which resulted in positive results [35]. These reports underscore the need to request quantitative or higher-dilution RPR when syphilis is strongly suspected, to avoid missed diagnoses from prozone reactions.

### 4.6. Change in the RPR Titer After Treatment

A four-fold or greater decrease in the RPR titer is used to assess treatment efficacy [18,30]. In this study, nine cases (64.3%) showed such a decrease, while five (35.7%) decreased by less than four-fold. In two cases (nos. 6, 10), the follow-up period was <2 months; thus, changes in RPR titers could not be adequately evaluated. Another case (no. 5) involved a patient in their 10s with early syphilis and a baseline RPR titer of 1:1, which became negative after treatment, making it difficult to assess a four-fold decrease. This aligns with a previous study showing that male (our patient was female), younger patients, or those with a baseline titer ≤ 1:2 or ≥1:64 or early syphilis were more likely to have nontreponemal antibody titers become negative or decrease four-fold after treatment [36]. The remaining two patients (nos. 11, 16) were elderly females with no chance of infection within 1 year, consistent with that report.

### 4.7. Follow-Up

In Japan, follow-up of serum antibody titers for approximately 1 year after treatment is recommended [18]. The Centers for Disease Control and Prevention recommends follow-up at 6 and 12 months for early syphilis and at 6, 12, and 24 months for latent syphilis [19]. At our hospital, only half of the cases were appropriately followed, while seven were not. Reasons for inadequate follow-up included short follow-up due to interrupted or terminated visits, no antibody test follow-up, and no quantitative RPR follow-up. The most common was short follow-up, seen in five of seven cases. In early syphilis, four cases (66.7%) had inadequate follow-up, all with RPR monitoring < 1 year. These patients stopped visits or completed quantitative RPR testing after both RPR decline and lesion improvement. No treatment failures or relapses occurred during follow-up. Reports have documented cases where follow-up of clinical findings and RPR led to relapse or reinfection detection and timely treatment [37,38]. Follow-up should be ensured for a sufficient period after treatment for early detection of treatment failure and reinfection. Therefore, it is important to clearly explain to patients the necessity of follow-up and to emphasize that they should continue hospital visits until their physician determines that further follow-up is unnecessary, even after RPR titers have declined and lesions have improved. Furthermore, physicians should regularly perform clinical assessments and quantitative RPR testing during follow-up.

### 4.8. Limitations

Although this study has the strength of comprehensively evaluating patients with syphilis over many years, it also has limitations. First, as it was conducted at a single hospital, the number of participants was small. Only 23 treated patients may limit generalizability. Second, there was departmental bias in requests for syphilis antibody testing, which influenced the results. Third, the study included some patients who were not assessed by infectious disease specialists. In contrast, the 23 treated cases underwent retrospective chart reviews conducted by infectious disease specialists. Fourth, referral bias may exist, as this study was conducted at a highly specialized hospital and the cases may not represent those in community settings. Fifth, this study focused on syphilis testing patterns and the clinical characteristics of the treated group; consequently, the follow-up status of the untreated group remains unknown.

## 5. Conclusions

This study aimed to clarify the clinical and laboratory characteristics of patients with syphilis diagnosed at a university hospital. Our findings show that syphilis can be diagnosed not only in urology, dermatology, and obstetrics and gynecology but also in ophthalmology and neurology, particularly in highly specialized medical institutions. The most frequently observed lesion was uveitis, underscoring the importance of including syphilis in the differential diagnosis of unexplained uveitis. Treponemal antibody tests show higher sensitivity than nontreponemal antibody tests in early syphilis, and clinicians must remain alert to the possibility of false-negative RPR results, including those from the prozone phenomenon. Accurate interpretation of serology and appropriate follow-up are essential for evaluating treatment efficacy and detecting relapse or reinfection early. In conclusion, especially in highly specialized medical institutions, clinicians should always consider the possibility of syphilis across diverse symptoms and departments, perform comprehensive testing, and maintain appropriate follow-up to improve patient outcomes.

## Figures and Tables

**Table 1:** Sensitivity, specificity for detecting treated patients, and positivity rate by treponemal antibody and RPR.

	Category	Treated	Not Treated	Total
**Treponemal Antibod** **y**	**Positive**	23 (100)	100 (1.1)	123 (1.3)
	**Negative**	0 (0)	9022 (98.9)	9022 (98.7)
	**Total**	23	9122	9145
**RPR**	**Positive**	18 (78.3)	98 (1.1)	116 (1.3)
	**Negative**	5 (21.7)	9024 (98.9)	9029 (98.7)
	**Total**	23	9122	9145

The numbers in the table represent the number (%) of patients. The sensitivity, specificity and positivity rate in treponemal antibody test were 100% (23/23), 98.9% (9022/9122) and 1.3% (123/9145), respectively. The sensitivity, specificity and positivity rate in RPR were 78.3% (18/23), 98.9% (9024/9122) and 1.3% (116/9145), respectively. RPR, rapid plasma reagin.

**Table 2:** Characteristics of the treated patients.

Characteristic	All Patients *n* = 23 No. (%)	Early Syphilis *n* = 8 No. (%)	Late Syphilis *n* = 5 No. (%)	Ocular Syphilis *n* = 8 No. (%)	Neuro Syphilis *n* = 4 No. (%)
**Sex**					
Male	14 (60.9)	5 (62.5)	2 (40.0)	5 (62.5)	2 (50.0)
Female	9 (39.1)	3 (37.5)	3 (60.0)	3 (37.5)	2 (50.0)
**Age**					
Male, median (IQR)	51.5 (46.8–57.0)	58.0 (46.0–67.0)	51.5 (50.8–52.3)	49.0 (44.0–50.0)	43.0 (37.5–48.5)
Female, median (IQR)	44.0 (35.0–73.0)	17.0 (17.0–30.5)	78.0 (60.0–79.5)	55.0 (45.0–64.0)	64.0 (59.5–68.5)
**Department**					
Ophthalmology	7 (30.4)	0	0	7 (87.5)	2 (50.0)
Dermatology	4 (17.4)	4 (50.0)	0	0	0
Obstetrics and gynecology	2 (8.7)	1 (12.5)	1 (20.0)	0	0
Urology	2 (8.7)	2 (25.0)	0	0	0
Neurology	2 (8.7)	0	1 (20.0)	1 (12.5)	1 (25.0)
Nephrology	2 (8.7)	0	1 (20.0)	0	0
Rheumatology	1 (4.3)	0	1 (20.0)	0	0
Orthopedic surgery	1 (4.3)	0	1 (20.0)	0	0
Oral and maxillofacial surgery	1 (4.3)	1 (12.5)	0	0	0
Psychiatry	1 (4.3)	0	0	0	1 (25.0)
**Comorbidity**					
Hypertension	6 (26.1)	0	2 (40.0)	3 (37.5)	2 (50.0)
Liver disease	4 (17.4)	1 (12.5)	0	2 (25.0)	1 (25.0)
Heart disease	4 (17.4)	2 (25.0)	2 (40.0)	0	0
Brain and Cerebrovascular disease	4 (17.4)	1 (12.5)	1 (20.0)	2 (25.0)	2 (50.0)
Diabetes	3 (13.0)	0	1 (20.0)	2 (25.0)	2 (50.0)
Malignant tumor	1 (4.3)	0	1 (20.0)	0	0
None	4 (17.4)	0	1 (20.0)	2 (25.0)	1 (25.0)
**History of STI**	4 (17.4)	2 (25.0)	0	2 (25.0)	2 (50.0)
**Phase**					
Early syphilis	8 (34.8)	8 (100)	0	0	0
Primary	3 (13.0)	3 (37.5)	0	0	0
Secondary	4 (17.4)	4 (50.0)	0	0	0
Early latent	1 (4.3)	1 (12.5)	0	0	0
Late syphilis	5 (21.7)	0	5 (100)	0	0
Tertiary	1 (4.3)	0	1 (20.0)	0	0
Unknown duration	4 (17.4)	0	4 (80.0)	0	0
Ocular syphilis	8 (34.8)	0	0	8 (100)	3 (75.0)
Neurosyphilis	4 (17.4)	0	0	3 (37.5)	4 (100)
Unknown	1 (4.3)	0	0	0	0
**Lesion**					
Patient with lesion	17 (73.9)	7 (87.5)	1 (20.0)	8 (100)	4 (100)
Uveitis	6 (26.1)	0	0	6 (75.0)	1 (25.0)
Optic nerve lesion	5 (21.7)	0	0	5 (62.5)	2 (50.0)
Neurosyphilis	4 (17.4)	0	0	3 (37.5)	4 (100)
Genital papules and induration and hard chancre	4 (17.4)	3 (37.5)	0	1 (12.5)	0
Retinal lesion	3 (13.0)	0	0	3 (37.5)	0
Rash	3 (13.0)	2 (25.0)	0	1 (12.5)	0
Condyloma latum	2 (8.7)	2 (25.0)	0	0	0
Oral mucositis	1 (4.3)	1 (12.5)	0	0	0
Inguinal lymphadenopathy	1 (4.3)	1 (12.5)	0	0	0
Alopecia	1 (4.3)	1 (12.5)	0	0	0
Tabes dorsalis	1 (4.3)	0	1 (20.0)	0	0
General paresis	1 (4.3)	0	0	0	1 (25.0)
None	5 (21.7)	1 (12.5)	4 (80.0)	0	0
Unknown	1 (4.3)	0	0	0	0
**Complication of** **O** **ther STI**					
Patient with other STI	1 (4.3)	0	0	1 (12.5)	0
HIV infection	1 (4.3)	0	0	1 (12.5)	0
None	22 (95.7)	8 (100)	5 (100)	7 (87.5)	4 (100)
**Treatment**					
Amoxicillin	16 (69.6)	8 (100)	4 (80.0)	4 (50.0)	0
1500 mg/d 4w Orally	4 (17.4)	2 (25.0)	1 (20.0)	1 (12.5)	0
1500 mg/d ≥ 5w Orally	5 (21.7)	3 (37.5)	1 (20.0)	1 (12.5)	0
Other	7 (30.4)	3 (37.5)	2 (40.0)	2 (25.0)	0
Penicillin	5 (21.7)	0	0	4 (50.0)	4 (100)
24 MU/d 2w iv	3 (13.0)	0	0	3 (37.5)	2 (50.0)
18 MU/d 2w iv	1 (4.3)	0	0	1 (12.5)	1 (25.0)
Other	1 (4.3)	0	0	0	1 (25.0)
Treatment for lightning pain	1 (4.3)	0	1 (20.0)	0	0
Unknown	1 (4.3)	0	0	0	0
**Symptom and** **Lesion after** **Treatment**					
Recovery	10 (43.5)	5 (62.5)	0	5 (62.5)	1 (25.0)
None	5 (21.7)	1 (12.5)	4 (80.0)	0	0
Consistency	3 (13.0)	0	1 (20.0)	1 (12.5)	2 (50.0)
Not applicable *	5 (21.7)	2 (25.0)	0	2 (25.0)	1 (25.0)
**Follow-up**	*n* = 15	*n* = 6	*n* = 2	*n* = 7	*n* = 3
Appropriate	8 (53.3)	2 (33.3)	1 (50.0)	5 (71.4)	3 (100)
Inappropriate	7 (46.7)	4 (66.7)	1 (50.0)	2 (28.6)	0

Data are presented as numbers (%) unless indicated otherwise. * Five patients could not be evaluated for symptoms and lesions owing to transfer, interrupted visits, or complications from other diseases. IQR, interquartile range; STI, sexually transmitted infection; HIV, human immunodeficiency virus; d, day; w, weeks; MU, million units; iv, intravenous.

**Table 3:** Results of treponemal antibody test and RPR.

The Results of Tests	All Patients *n* = 23 No. (%)	Early Syphilis *n* = 8 No. (%)	Late Syphilis *n* = 5 No. (%)	Ocular Syphilis *n* = 8 No. (%)	Neuro Syphilis *n* = 4 No. (%)
**Initial** **T** **reponemal** **T** **est**					
Median * (IQR)	71.1 (20.3–>100.0)	68.5 (13.7–>100.0)	41.7 (1.5–70.6)	92.8 (68.4–>100.0)	78.4 (20.3–>100.0)
(Range)	(1.2–>100.0)	(1.2–>100.0)	(1.4–82.4)	(20.3–>100.0)	(20.3–>100.0)
**Initial RPR**					
Positive	18 (78.3)	6 (75.0)	3 (60.0)	7 (87.5)	4 (100)
Negative	5 (21.7)	2 (25.0)	2 (40.0)	1 (12.5)	0
Change to positive	1 (4.3)	1 (12.5)	0	0	0
**RPR Titer before** **Treatment**	*n* = 16	*n* = 6	*n* = 3	*n* = 6	*n* = 4
Average	39.6	38.8	2.0	60.3	26.5
Median (IQR)	20.0 (2.0–64.0)	18.0 (4.0–56.0)	2.0 (2.0–2.0)	48.0 (14.0–112.0)	20.0 (6.5–40.0)
(Range)	(1.0–128.0)	(1.0–128.0)	(2.0–2.0)	(2.0–128.0)	(2.0–64.0)
**RPR Titer after** **Treatment**	*n* = 14	*n* = 5	*n* = 2	*n* = 6	*n* = 4
Decrease	11 (78.6)	5 (100)	0	5 (83.3)	3 (75.0)
4-fold or greater decrease	9 (64.3)	3 (60.0)	0	5 (83.3)	3 (75.0)
2-fold decrease	1 (7.1)	1 (20.0)	0	0	0
1:1 to negative	1 (7.1)	1 (20.0)	0	0	0
No change	2 (14.3)	0	1 (50.0)	1 (16.7)	1 (25.0)
Increase	1 (7.1)	0	1 (50.0)	0	0
2-fold increase	1 (7.1)	0	1 (50.0)	0	0
**Time to 2-fold** **decrease**	*n* = 8	*n* = 4	*n* = 0	*n* = 4	*n* = 1
Median (IQR)	32 (26.8–70.8)	32 (29.3–53.5)	NA NA	41.5 (26.8–70.8)	NA NA
(Range)	(21.0–118.0)	(21.0–118.0)	NA	(23.0–118.0)	NA
**Time to 4-fold or** **Greater Decrease**	*n* = 8	*n* = 3	*n* = 0	*n* = 5	*n* = 2
Median (IQR)	143.5 (98.5–169.5)	161.0 (143.5–164.5)	NA NA	111.0 (61.0–174.0)	NA NA
(Range)	(49.0–186.0)	(126.0–168.0)	NA	(49.0–186.0)	NA

Data are presented as numbers (%) unless indicated otherwise. * The median was calculated with values above 100 as 100. IQR, interquartile range; RPR, rapid plasma reagin; NA, not applicable.

## Data Availability

The original contributions presented in this study are included in the article and Supplementary Material; further inquiries can be directed to the corresponding author.

## Supplementary Materials

The following supporting information can be downloaded at: https://www.mdpi.com/article/10.3390/pathogens14090892/s1, Table S1: List of the treated patients.

## Abbreviations

The following abbreviations are used in this manuscript:

CIA	Chemiluminescence immunoassay
CLEIA	Chemiluminescent enzyme immunoassay
COVID-19	Coronavirus disease 2019
EIA	Enzyme immunoassay
HIV	human immunodeficiency virus
IQR	Interquartile range
RPR	Rapid plasma reagin
STI	Sexually transmitted infection
TP	Treponemal antibody test
